# Epistasis Analysis Goes Genome-Wide

**DOI:** 10.1371/journal.pgen.1006558

**Published:** 2017-02-16

**Authors:** Jianzhi Zhang

**Affiliations:** Department of Ecology and Evolutionary Biology, University of Michigan, Ann Arbor, Michigan, United States of America; National Institute of Genetics, JAPAN

Epistasis, a term coined by William Bateson in 1909 [1], refers to the interdependence of mutations in their phenotypic effects. Let the phenotypic value of a trait relative to that of the wild type be *f*_A_ and *f*_B_ for mutants A and B, respectively, and let the phenotypic value of the corresponding double mutant be *f*_AB_. Although variation exists, epistasis is usually defined by ε = *f*_AB_ − *f*_A_*f*_B_ and is said to be positive when ε > 0 and negative when ε < 0.

Life would have been much simpler, and perhaps even boring, if epistasis were completely absent. In reality, however, epistasis abounds, rendering biology full of surprises and complexity. For instance, a commonly encountered type of epistasis is synthetic lethality, where simultaneously deleting two genes from the genome of a normal organism is lethal despite the fact that deleting each of them separately is viable. Using the notation introduced above, we can describe synthetic lethality by *f*_A_ > 0, *f*_B_ > 0, and *f*_AB_ = 0; consequently ε *<* 0. A simple mechanistic explanation of synthetic lethal epistasis is that the two genes investigated are functionally similar and hence redundant. Clearly, studying epistasis helps us to understand the functional relationship between genes, which is critical to uncovering the inner workings of biological systems. Epistasis can explain why hybrids between species are typically inviable or infertile [2] and is believed to underlie the intriguing phenomenon that some human disease-causing mutations are fixed in other species with no apparent detriment [3]. Furthermore, epistasis is assumed in many evolutionary theories. For example, the mutational deterministic hypothesis of the evolution of sexual reproduction [4] and the hypothesis of reduction in mutational load by truncation selection against deleterious mutations [5] both depend on overall negative epistasis. Thus, verifying these hypotheses requires confirming the prevalence of negative epistasis.

Epistasis is typically detected by demonstrating the inequality between *f*_AB_ and *f*_A_*f*_B_ or some consequences of this inequality. The advent of next-generation sequencing and other genomic technologies is quickly enlarging the scale of epistasis studies. Of special significance is the recent completion of the yeast genetic interaction map, which includes nearly all 36 million epistasis values for pairs of ~6,000 yeast genes estimated from the growth rates of single- and double-gene–deletion mutants [6]. Although this map provides unprecedented data of epistasis between null mutations of different genes, it offers no information on the epistasis between mutations at different sites within a gene or that between non-null mutations in different genes.

Complementing the above coarse-grained epistasis map are nucleotide-resolution epistasis maps of individual genes or segments of genes [7–10]. For instance, Li et al. synthesized a yeast tRNA gene with error, creating all possible single-point mutation variants of the gene as well as tens of thousands of variants with multiple mutations [7]. They then used a high-throughput method to measure the fitness of yeast strains, each carrying a variant tRNA gene at the place of the endogenous gene, and estimated epistasis between mutations. Interestingly, negative epistasis was found to be more prevalent than positive epistasis [7]. In principle, such a map can be constructed for every gene in the yeast genome to acquire a general picture of epistasis.

Even with both the coarse- and fine-grained epistasis maps, we still do not have epistasis data between non-null mutations of different genes, which constitute the largest part of a complete nucleotide-resolution epistasis map of a genome. Take yeast as an example: this largest part contains potentially 10^14^ interactions. Obviously, determining the complete epistasis map is an enormous challenge.

In this issue of *PLOS Genetics*, Skwark and colleagues [11] harnessed population genomic data to approach this challenge. Specifically, they developed a computational method termed genomeDCA to detect epistasis using genotype and allele frequencies estimated from genome sequences of thousands of individuals of the same species. Their method is a modification of direct-coupling analysis (DCA), a statistical method in structural biology for predicting direct residue contacts within and between proteins [12,13]. What is the similarity between physical residue contacts and epistasis that allows the borrowing of DCA for identifying epistasis? Well, if two sites contact in protein structure, the amino acid that sits at one site likely impacts what amino acids can occupy the other site. One can imagine the scenario in which amino acid A at site 1 interacts well with amino acid B at site 2, but when A is mutated to A′ or when B is mutated to B′, the interaction is disrupted. However, when both residues are mutated, the interaction may be restored, resulting in ε = *f*_A′B′_ − *f*_A′B_*f*_AB′_ > 0, where *f* is the strength of interaction relative to that between A and B. If fitness increases with the interaction strength, one would frequently observe the genotype of AB or A′B′ at the two sites but rarely encounter AB′ or A′B when many species are examined. Thus, protein sequences from many species provide information about residue contacts as well as epistasis. This idea forms the basis of DCA in structural biology, although the actual statistical analysis is more sophisticated due to a number of confounding factors such as the phylogenetic nonindependence of protein sequences.

**Fig 1:**
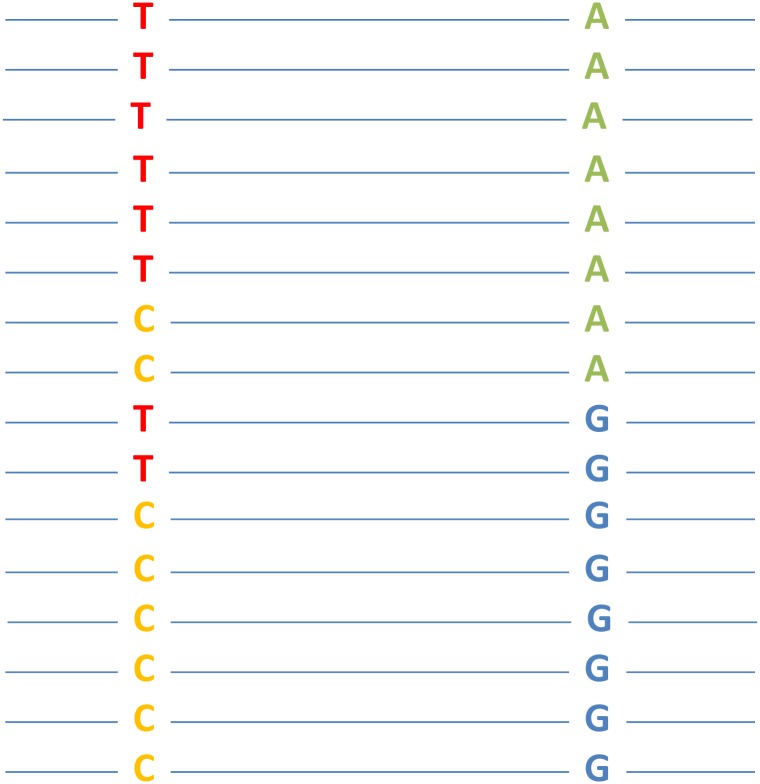
A hypothetical example of epistasis between two nucleotide sites that is detectable by genomeDCA. Each line represents a (haploid) genome sequence from an individual, where two freely recombining nucleotide positions are shown. Each position has two states in the population. In this example, the genotype frequencies of TA and CG are both 6/16, exceeding the expectation (0.5 × 0.5 = 4/16) from allele frequencies under no epistasis. Should this discrepancy be statistically significant, epistasis between the two sites is detected (genotypes TA and CG are fitter than TG and CA).

GenomeDCA is similar to DCA but is applied to genome sequences of a large number of conspecifics. When there is no population structure and when all individuals are recombining freely, linkage disequilibrium between two nucleotide sites should approach zero unless the relative fitness of the double mutant does not equal the multiplication of those of the corresponding single mutants (Fig 1). Hence, detection of linkage disequilibrium between two sites indicates epistasis. Of course, sites that are close in chromosomal location may be at linkage disequilibrium due to limited recombination. Thus, genomeDCA should be applied to sites that are sufficiently far apart on the same chromosome or located on different chromosomes. The main advantage of genomeDCA over traditional epistasis analyses is its genomic scale and its ability to test epistasis for many mutation pairs from one large set of genome sequences. The chief disadvantage is that, given the limited number of conspecifics sampled, only a tiny fraction of all possible mutations is observed, which dictates the number of mutation pairs for which epistasis can be evaluated by genomeDCA. In other words, one may not be able to test epistasis between a predetermined pair of mutations, because these mutations may not be present in the genome sequences or may not be sufficiently common to guarantee statistical power. This situation applies to many deleterious mutations, which are either not present or have low frequencies even in reasonably large samples. Nevertheless, depending on the purpose of the epistasis analysis, one could argue that it is the epistasis between observed mutations that is most relevant. In this regard, genomeDCA detects the most relevant epistasis. Another limitation of genomeDCA is that it detects only fitness epistasis, whereas traditional methods can detect epistasis in other traits.

Skwark et al. applied genomeDCA to two large population genomic datasets: 3,156 genomes of *Streptococcus pneumoniae* isolates and 3,442 genomes of *Streptococcus pyogenes* isolates. Their results are biologically interesting. For example, they detected over 5,000 epistatic interactions in the former dataset, over three quarters of which are between sites in three genes (*pbp2x*, *pbp1a*, and *pbp2b*) that confer resistance to beta-lactam antibiotics. With the precipitous drop of the cost of DNA sequencing and rapid accumulation of population genomic data of humans, human pathogens, and genetic model organisms, genomeDCA promises to offer a cost-effective survey of epistasis at the genomic scale. As epistasis data accrue, one can start looking for general patterns and underlying mechanisms of epistasis [14], which will ultimately aid our understanding of the organizing principles of biological systems.
